# Conservation Genetics of the Asian Giant Soft-Shelled Turtle *(Pelochelys cantorii)* with Novel Microsatellite Multiplexes

**DOI:** 10.3390/ani12243459

**Published:** 2022-12-08

**Authors:** Minmin Xie, Chen Chen, Yakun Wang, Wei Li, Lingyun Yu, Xiaoyou Hong, Xinping Zhu

**Affiliations:** 1 Key Laboratory of Tropical & Subtropical Fishery Resource Application & Cultivation of Ministry of Agriculture, Pearl River Fisheries Research Institute, Chinese Academy of Fishery Sciences, Guangzhou 510380, China; 2 College of Wuxi Fisheries, Nanjing Agricultural University, Wuxi 214081, China

**Keywords:** *Pelochelys cantorii*, microsatellite, conservation groups, genetic diversity, genetic structure, nondestructive sampling

## Abstract

**Simple Summary:**

*Pelochelys cantorii* is critically endangered and rarely seen in the wild, and only 13 adults are being kept in captivity in China. For the purpose of reinforcing the conservation and management of *P. cantorii*, the Pearl River Fisheries Research Institute (Chinese Fishery Academy of Sciences) successfully bred more than 800 turtles from 2015 to 2020. In this study, we developed and characterized 10 simple sequence repeat markers from the RNA transcriptome of *P. cantorii*, established two multiplex PCR systems, and obtained the genetic structure and genetic diversity of the artificially conserved population. The aim was to obtain viable second-generation *P. cantorii* with the highest genetic diversity to implement population recovery plans for this species.

**Abstract:**

To understand the genetic structure of the protected turtle species *Pelochelys cantorii* we used transcriptome data to design more than 30,000 tri- and tetranucleotide repeat microsatellite primer pairs, of which 230 pairs were used for laboratory experiments. After two screenings, only 10 microsatellite markers with good specificity, high amplification efficiency, and polymorphisms were obtained. Using the selected primers, two multiplex PCR systems were established to compare and analyze the genetic diversity of artificially assisted breeding generations from four parents (two females and two males) continuously bred over two years. A total of 25 alleles were detected among the 10 microsatellite loci of the offspring. The polymorphism information content (PIC) was 0.313–0.674, with an average of 0.401, among which two loci were highly polymorphic (PIC ≥ 0.5). The number of alleles was 2–5 and the number of effective alleles was 1.635–3.614. The observed heterozygosity was 0.341–0.813, with an average of 0.582, whereas the average expected heterozygosity was 0.389–0.725, with an average of 0.493. Moreover, on the basis of Nei’s genetic distance and the Bayesian clustering algorithm, the 182 offspring were divided into two subgroups, and the results corresponded to the two maternal lines. This is the first study to investigate the molecular markers of *P. cantorii*, and the results obtained can be used to protect genetic resources and provide a genetic basis for the design of population recovery plans.

## 1. Introduction

The Asian giant soft-shelled turtle (*Pelochelys cantorii*) belongs to the order Testudines (family: *Trionychidae*), one of the largest inland aquatic turtle species. It is an important indicator of ecological health in the Pearl River and the south of the Yangtze River in China. This species also has a long history and is of great scientific value in paleogeography and paleontological evolution. In the past, *P. cantorii* was widely distributed in southeastern China, but due to excessive economic development, its habitat has continuously deteriorated, and its population has been greatly reduced. Currently, *P. cantorii* is critically endangered and rarely seen in the wild, and only 13 adults are kept in captivity [1]. To strengthen its protection policy, China listed the turtle as a key aquatic wildlife protection animal at a national level in 1989. The World Conservation Union approved *P. cantorii* as an endangered species in 2000, and it was later added to a page in Appendix II of the Convention on International Trade in Endangered Species of Wild Fauna and Flora treaty in 2003.

For the purpose of reinforcing the conservation and management of *P. cantorii*, the Pearl River Fisheries Research Institute (Chinese Fishery Academy of Sciences) successfully bred more than 800 turtles from 2015 to 2020 using four sexually mature turtles (two females and two males). Twenty healthy *P. cantorii*, aged 45 years and weighing 1.5–2 kg, were selected for a rewilding adaptive protection test in 2020 [2]. Nonetheless, the genetic structure and diversity of the artificially conserved population are still unclear; therefore, scientific management is urgently needed to obtain viable second-generation *P. cantorii* with high genetic diversity and restore the wild population through artificial propagation and release.

Microsatellite markers are codominant markers with a high degree of polymorphism [3,4,5]. They are effective molecular markers in the field of population genetic diversity detection as well as genetic map construction [6,7,8]. With the rapid development of high-throughput sequencing technology, it is now easier and cheaper to screen microsatellite markers, which also promotes their wide application. Microsatellite markers have been successfully applied in various studies on aquatic biology [9,10,11,12] and testudines [13,14,15,16,17,18].

In this study, we obtained transcriptomic data for *P. cantorii*. The main goal was to develop microsatellite multiplexes for *P. cantorii* to evaluate the genetic diversity and structure of artificially assisted breeding generations and to design the most feasible population recovery plan for this species.

## 2. Materials and Methods

### 2.1. Experimental Materials

The experimental materials were obtained from a *P. cantorii* breeding and protection base in Gaoming, China, which maintains four parents (two females and two males). There were a total of 182 offspring (103 individuals born in 2019 and 79 in 2020). All of them underwent artificial incubation, whereafter umbilical cords were collected after hatching. The umbilical cords were soaked in anhydrous ethanol and stored at −20 °C.

Permission for this work was obtained from the relevant staff at the Gaoming breeding and protection base. We only used the umbilical cord as experimental material, which can naturally fall off, to prevent influencing individual biological behaviors.

### 2.2. Genomic DNA Extraction

Umbilical cords were cut to a mung bean size (approximately 30 μg), and a MicroElute Genomic DNA Kit (OMEGA Bio-Tek, Inc., Norcross, GA, USA) was used for DNA extraction according to the manufacturer’s specifications. The optical density (DNA absorbance ratio) and concentration of the extracted DNA were measured using a Nano Q microspectrophotometer (BoAo, USA). Then, 1% agar gel electrophoresis was performed to test DNA integrity. The DNA solutions were stored at −20 °C until use.

### 2.3. Design and Screening of Microsatellite Primers

Using transcriptome data produced in our laboratory (unpublished), tri- and tetranucleotide repeat microsatellite sequences were identified, and primers were designed using Primer Premier 5.0 [19]. Thereafter, eight turtle samples were randomly selected to preliminarily detect primer specificity and polymorphism at the optimum annealing temperature. The selected primers were combined to construct a multiplex PCR system. Notably, the lengths of the PCR amplification products of the same joint primers did not overlap. During primer synthesis, different joints (PET, VIC, and NED) were added at the 5′ end of each forward primer. In addition, the synthesized PET, NED, and VIC sequences were labeled with red, black, and green fluorescence, respectively (Table 1).

Before PCR amplification, all primers were diluted to 10 μmol/L and mixed at a 1:40 ratio for each pair of forward and reverse primers. All three fluorescent connectors were diluted to 20 μmol/L. The established 10 μL multiplex PCR system comprised the following: 5 μL Applied Biosystems Multiplex PCR Master Mix; 2 μL mixed primers (forward primer and reverse primer); 0.2 μL fluorescent connector, 1.8 μL deionized water; 30–150 μmol/L DNA; 1 μL. The amplification procedure was as follows: initial denaturation at 94 °C for 5 min; denaturation at 94 °C for 30 s, annealing at 60 °C for 45 s, and extension at 72 °C for 70 s (24 cycles); denaturation at 94 °C for 30 s, annealing at 53 °C for 40 s, and extension at 72 °C for 30 s (eight cycles); final extension at 72 °C for another 10 min. A 2 μL volume of PCR product was mixed with 8 μL of molecular weight marker (GeneScan 500 LIZ)™ and a deionized formamide mixture (1:100). After further incubation for 5 min at 95 °C, the mixture was cooled on an ice plate for rapid denaturation. Thereafter, capillary electrophoresis was performed using an Abi 3130 multifunctional genetic analyzer (Applied Biosystems, Waltham, MA, USA). Finally, Peak Scanner Software v1.00 was used for genotyping.

### 2.4. Data Analysis

POPGENE v1.32 [20] was used to calculate the number of alleles (N_a_), effective alleles (A_e_), observed heterozygosity (H_o_), expected heterozygosity (H_e_), and Shannon diversity index of the microsatellite loci. The polymorphism information content (PIC) of the microsatellite loci was calculated using CERVUS v3.0 [21]. Calculation of the inbreeding coefficient (F_IS_) among turtles was performed using GenAlEx 6 [22].

POPGENE v1.32 was used to treat each sample as a population to calculate Nei’s standard genetic distance for each turtle, and dichotomous difference clustering (evolutionary tree) was constructed with MEGA 5.0 [23] to obtain the classification relationship between each of them.

Structure v2.3.4 [24] was used to simulate the number of subgroups, and the Bayesian clustering algorithm was used to calculate the clustering status and blood composition of each turtle. Mapping was obtained by CLUMPP; K values were selected from 1–7, and each value was repeated six times, preheated 50,000 times, discarded, and followed by 100,000 formal calculations. The package structure results (K = 1–7) were submitted to http://taylor0.biology.ucla.edu/structureHarvester/ (accessed on 6 January 2020) and concluded as the best K value.

## 3. Results

### 3.1. DNA Extraction and Quality Control

**Figure 1:**
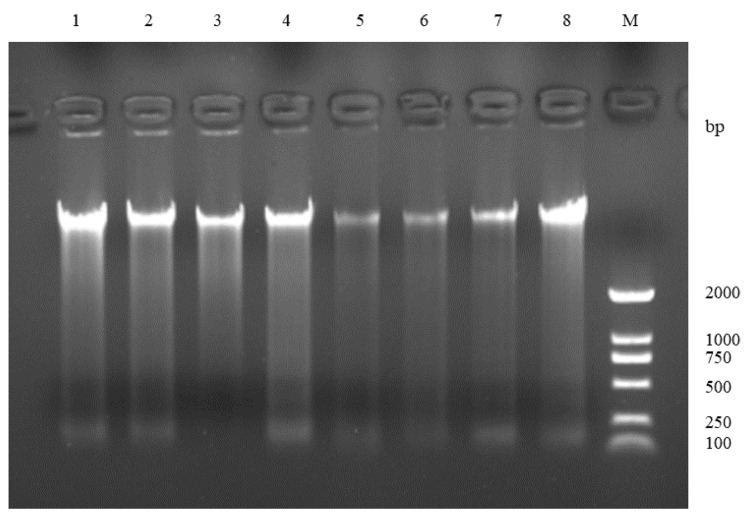
Genomic DNA gel electrophoresis of some samples from *Pelochelys cantorii*.

DNA integrity was evaluated using 1% agarose gel electrophoresis. When the band pattern was clear and bright (Figure 1), DNA integrity was considered good, the original figure is shown in Figure S1. The 260/280 ratio of DNA ranged from 1.82–1.92, indicating that DNA integrity was good and the purity was high, which met the requirements of the subsequent experiments.

### 3.2. Design and Screening of Microsatellite Primers

More than 30,000 tri- and tetranucleotide repeat microsatellite primer pairs were designed. Trinucleotide repeat microsatellite primers comprised 67.73% of the total, and the number of (AAT)_n_ sequence motifs was the largest, accounting for 6.00%. Tetranucleotide microsatellite primers consisted of 32.27% of the total, and (AAAG)_n_ sequence motifs were the most numerous, accounting for 6.67%. Among these, 230 primer pairs were randomly selected. In total, 153 pairs were amplified to form stable bands after the first screening. Eight individual samples were randomly selected for detection, and 10 pairs were polymorphic, with a polymorphism ratio of 6.53%. Relevant information on the 10 microsatellite primer pairs is shown in Table 2.

### 3.3. Construction of Multiplex PCR

**Figure 2:**
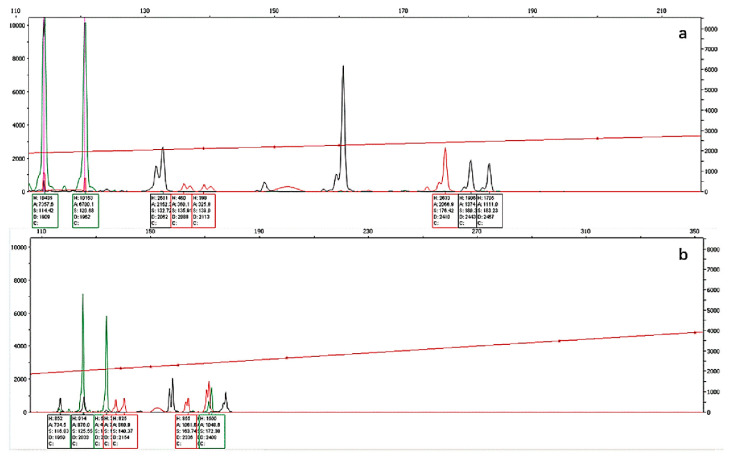
Electrophoretic patterns determined after multiplex PCR using (**a**) group 1 and (**b**) group 2 primers for individual No. 24.

**Figure 3:**
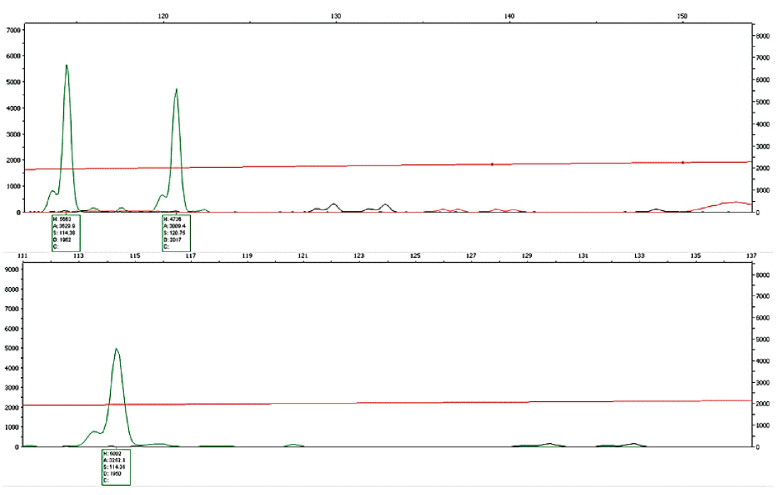
Electrophoretic patterns of Xm17 for random turtles. The number of isotopic genes indicates whether the individual was heterozygous at this site; the bimodal and unimodal distributions represent heterozygosity and homozygosity, respectively.

By optimizing the parameters of multiplex PCR, two groups of microsatellite multiplex PCR systems were established, each containing five microsatellite loci. The specific parameters of the system are shown in Table 3 and Table 4, and the partial electrophoresis patterns of each multiplex PCR group are shown in Figure 2 and Figure 3.

### 3.4. Analysis of Population Genetic Diversity

All 10 microsatellite markers used in both PCR combinations amplified stable and clear DNA bands in the 182 turtle samples. The band size was 114–190 bp, and the number of alleles detected at each site was 2–5. The PIC was 0.313–0.674, of which two were highly polymorphic (PIC ≥ 0.5) and eight were intermediate polymorphic markers (0.25 ≤ PIC ≤ 0.5). The Shannon diversity index was 0.577–1.335, with an average value of 0.755. The F_IS_ was −0.611–0.310. The N_a_ in the 2019 samples was 2–5, with an average of 2.5. The N_e_ was 1.606–0.377, and H_o_ was 0.359–0.854, with an average of 0.609. The H_e_ was 0.379–0.737, and the mean was 0.498. The Shannon diversity index ranged from 0.565–1.353, with an average value of 0.764. The N_a_ in the 2020 samples was 2–4, with an average of 2.3. The N_e_ was 1.576–3.346 and the H_o_ was 0.317–0.760, with an average of 0.547. H_e_ was 0.368–0.706, with an average of 0.482. The Shannon diversity index was 0.552–1.291, with an average value of 0.731. The genotyping call rate of the 10 microsatellite loci (N = 182 individuals) ranged from 34.07% to 81.87%. The number of alleles and available alleles, H_o_, H_e_, and Shannon diversity index in 2019 were all higher than those in 2020. The statistical results of the observational data are shown in Table 5, Table 6, Table 7 and Table 8.

### 3.5. Genetic Relationship Analysis

**Figure 4:**
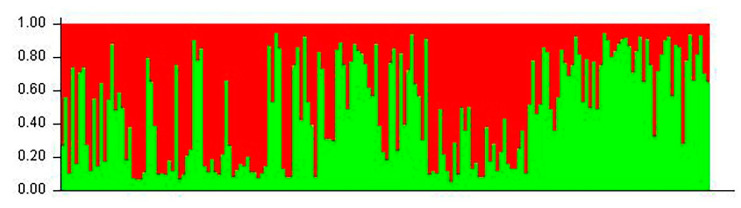
Individual clustering map based on cladograms. The individuals numbered 1–103 were F_1_ *Pelochelys cantorii* from 2019 and 104–182 were from 2020. Data were based on the Bayesian clustering algorithm.

**Figure 5:**
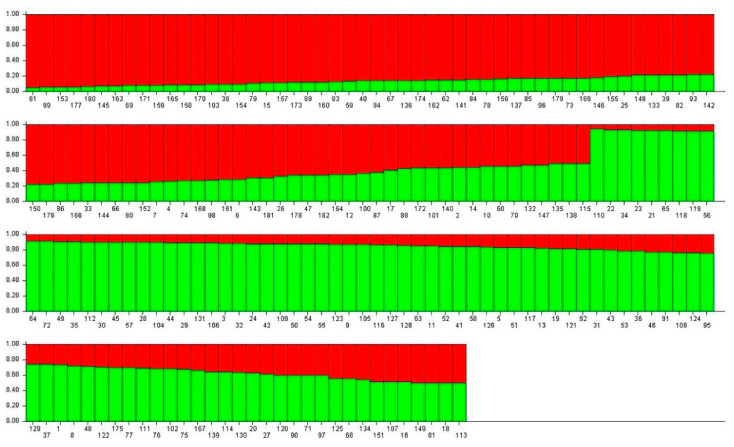
Population genetic structure when the subgroup value was K2 (K = 2). Data were based on the Bayesian clustering algorithm.

**Figure 6:**
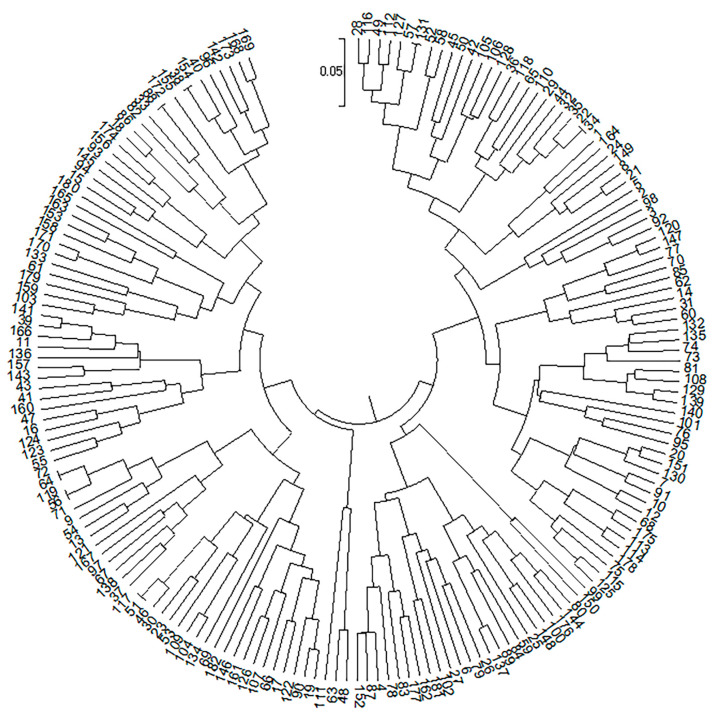
Degree of individual interbreeding when the subgroup value was 2 (K = 2). Data based on Nei’s genetic distance.

The genetic distance between each individual was calculated using POPGENE. The individual clustering diagram based on the evolutionary tree method using MEGA showed that the entire progeny population could be divided into two subgroups (Figure 2). Beyond that, it was also concluded that when the subgroup value was 2, it could be measured using Structure software. Each individual was represented by a vertical bar partitioned into segments according to the proportion of genome belonging to each of the clusters identified (K = 2) using Structure (displayed from left to right in the order of 1–183). The number of subgroups was also the closest to reality, and the degree of individual hybrids is shown in Figure 4, Figure 5 and Figure 6. Individuals numbered 1–103 were offspring from 2019 and 104–182 were offspring from 2020.

## 4. Discussion

### 4.1. Screening and Polymorphism of Microsatellite Markers

Yue et al. (2016) performed transcriptome sequencing of the reproductive tissues of *Cyprinus carpio* var. *singuonensis* and screened a large number of polymorphic microsatellite loci [25]. Twelve microsatellite markers obtained from an RNA transcriptome were constructed from blood cells of *Tachypleus tridentatus*, which could also be applied to the analysis of its genetic diversity [26]. According to Lindqvist et al. [27], microsatellite markers of tri- and tetranucleotide repeats are more suitable for the large-scale automatic analysis of fluorescent labels. Lu et al. [28] also showed that microsatellite markers with tri- and tetranucleotide repeats in *C. carpio* had more abundant polymorphisms. In vertebrate genomes, microsatellite tetranucleotides (GATA/AGAT) are the most common [29]; therefore, microsatellite enrichment of many species has subsequently been carried out using GATA/AGAT sequence motifs as a probe. In this study, more than 30,000 tri- and tetranucleotide repeat microsatellite primer pairs were selected and designed from the transcripts of *P. cantorii*. The AAAG_(n)_ sequence motif was the most prevalent, accounting for 6.67% of the total, whereas GATA and AGAT sequence motifs only represented 1.92% and 1.57%, respectively. This may be related to the study design criteria and species specificity.

### 4.2. Genetic Structure of the Soft-Shelled Turtle Population

Compared with other testudines, the population of *P. cantorii* observed in this study had fewer alleles. For example, genetic differentiation of *Geochelone nigra* using 10 microsatellite loci showed 12–37 alleles in each microsatellite seat in various groups, with an average of 21.1 [30]. In addition, the number of alleles determined for *Malaclemys terrapin* ranged from 8–14 in five microsatellite loci, which was an approximate average of 10.67 [31]. The number of alleles (A) at a single locus in *Mauremys mutica* ranged from 5–26, with an average of 14.190 [18].

Schultz et al. (2009) believed that a PIC > 0.5 is high, 0.5 > PIC > 0.25 is moderate, and a PIC < 0.25 is low. In this experiment, the number of alleles detected at each locus was 2–5 [32]. The PIC was 0.313–0.674, of which two were highly polymorphic (PIC ≥ 0.5). The Shannon diversity index was 0.7549. The H_o_ was similar to that of nine populations of *T. tridentatus* (0.46–0.57) and higher than that of the endangered *Cuora flavomarginata* (0.032–0.936), with an average value of 0.329 [33,34]. The data were also similar to the average H_o_ (0.512–0.627) of 218 individuals from four *Pelodiscus sinensis* populations [16]. These results indicate that the genetic diversity of *P. cantorii* is moderate. However, it is worth noting that the number of alleles and available alleles, H_o_, H_e_, Shannon diversity index, and other data from 2019 were all higher than those from 2020. The reasons for this may be the following: (1) the number of umbilical cord samples used varied—in 2019 and 2020, there were 103 and 79 samples, respectively; (2) these experiments took samples from the same four parents (two females and two males), and every parent mating combination was haphazard; (3) genetic diversity analysis was performed only on offspring from 2019 and 2020, which is a short sampling period.

In this experiment, MEGA and Structure software were used to cluster the conserved population. The results showed that the population could be divided into two subgroups, which was consistent with the actual situation of producing offspring from only two maternal parents. MEGA’s evolutionary tree results show that the population could be further divided into four subsets, consistent with the actual situation in which all four parents participate in reproductive activity and produce theoretical offspring groups (M_1_ × F_1_, M_1_ × F_2_, M_2_ × F_1_, and M_2_ × F_2_). The fact that the four subgroups had different numbers of progeny may be due to male competition during mating, including female selection preference, sperm motility difference, fixed collocation, and other factors. This phenomenon indicates that there are dominant individuals among the parents, but the emergence of such individuals may cause problems, such as migration and a reduction in genetic diversity in the offspring population. For the breeding of second-generation offspring in future strategies, individuals of different subgroups can be selected according to clustering data, and gene exchange within the population can be artificially mediated to prevent inbreeding decline.

## 5. Conclusions

The breeding period we used to develop a conservation population of *P. cantorii* was short, and sexually mature individuals are very rare in China. The Pearl River Fisheries Research Institute (Chinese Fishery Academy of Sciences) collaborating with Gaoming’s breeding center for *P. cantorii* has had no genotype supplementation from individuals of other regions in the short term. For all offspring derived from the four initial parents, germplasm resources are very limited, and the number of close relatives may increase in the future. Therefore, the introduction of sexually mature individuals from other regions may be an effective method to improve the level of genetic diversity, strengthen gene exchange among populations, and maintain the genetic potential of the population in prospective studies. At the same time, on the basis of molecular markers of parental genotype files and the genetic distances and relationships of the parents used to set up the scientific breeding program, we propose that it is possible to ensure that the offspring inherit the full genetic variation of the parents. This will allow them to produce offspring with rich genetic diversity and facilitate the supplementation of artificial breeding populations into the natural population.

## Figures and Tables

**Table 1:** Characterization of the three fluorescent joints.

Fluorescent Label	Joint Sequence	Fluorescence Color
PET	CACGACGTTGTAAAACGAC	Red
VIC	CAGGAACTCAGTGTGACACTC	Green
NED	CGACAGACAGTAAGGTCTCTG	Black

**Table 2:** *Pelochelys cantorii* primer sequences and amplification conditions.

Locus	Primer Sequence (5′-3′)	Repeat Motif	Annealing Temperature (°C)	Amplicon Size (bp)
Xm17	F-CACCACAGAGTGCATTGTCTTT R-AAGCCTTCTTTTAGCCATGTAGC	(TTG)_6_	60	114~120
Xm86	F-AAACTGGAAGAGTATCTTTGGGC R-CTGCCTTAGGTGTACTGGAGGAT	(AGC)_12_	60	174~190
Xm89	F-CAATTTAAACTGGCCAAAGACTG R-TAGGCCTTAGACTCATGCTGTTC	(GTTT)_5_	60	165~172
Xm99	F-TTTCTGCTCCTGCTCATCACTAC R-GTCCTCCTCCTCTGGAATGG	(GCT)_7_	61	117~126
Xm101	F-CTAGGGCCAGGAATCACTCAC R-CTTTCCCCTCTATGATGGTCTCT	(GACA)_5_	61	163~171
Xm106	F-TATCACTTGCAGGACCAAATTCT R-TAGGAATCACACATGCACAACTT	(ACC)_5_	60	180~183
Xm169	F-ACTGAAAATATGGAAAGGGGTGT R-CCAAGCACAGTCAGCAGATAATA	(ATA)_6_	60	129~132
Xm207	F-TCTGCCTTGGGTCACTTATTATC R-TGCAAAACAACATTTTCTGCTAA	(TTA)_9_	59.5	136~139
Xm209	F-GTACCACAGTGGCATTTCAGAAT R-CTCCTTTCTGTGGAAAATCTCG	(CAA)_7_	60.5	137~140
Xm225	F-GAAATCAGAGAACAGAGAGGCAA R-ATATAAGAATCAACCTGGACCCC	(AAC)_5_	60	124~136

**Table 3:** First group of multiplex PCR primers.

Locus	Primer Sequence	Joint Sequence	Fluorescent Label
Xm86	F-AAACTGGAAGAGTATCTTTGGGC R-CTGCCTTAGGTGTACTGGAGGAT	CACGACGTTGTAAAACGAC	PET
Xm207	F-TCTGCCTTGGGTCACTTATTATC R-TGCAAAACAACATTTTCTGCTAA	CACGACGTTGTAAAACGAC	PET
Xm106	F-TATCACTTGCAGGACCAAATTCT R-TAGGAATCACACATGCACAACTT	CGACAGACAGTAAGGTCTCTG	NED
Xm169	F-ACTGAAAATATGGAAAGGGGTGT R-CCAAGCACAGTCAGCAGATAATA	CGACAGACAGTAAGGTCTCTG	NED
Xm17	F-CACCACAGAGTGCATTGTCTTT R-AAGCCTTCTTTTAGCCATGTAGC	CAGGAACTCAGTGTGACACTC	VIC

**Table 4:** Second group of multiplex PCR primers.

Locus	Primer sequence (5′-3′)	Joint sequence (5′-3′)	Fluorescent Label
Xm101	F-CTAGGGCCAGGAATCACTCAC R-CTTTCCCCTCTATGATGGTCTCT	CACGACGTTGTAAAACGAC	PET
Xm209	F-GTACCACAGTGGCATTTCAGAAT R-CTCCTTTCTGTGGAAAATCTCG	CACGACGTTGTAAAACGAC	PET
Xm99	F-TTTCTGCTCCTGCTCATCACTAC R-GTCCTCCTCCTCTGGAATGG	CGACAGACAGTAAGGTCTCTG	NED
Xm89	F-CAATTTAAACTGGCCAAAGACTG R-TAGGCCTTAGACTCATGCTGTTC	CAGGAACTCAGTGTGACACTC	VIC
Xm225	F-GAAATCAGAGAACAGAGAGGCAA R-ATATAAGAATCAACCTGGACCCC	CAGGAACTCAGTGTGACACTC	VIC

**Table 5:** Genotyping call rate of 10 microsatellite DNA markers for the F_1_ population of *Pelochelys cantorii*.

Locus	Genotyping Number	Genotyping Call Rate
Xm86	149	81.87%
Xm207	62	34.07%
Xm106	94	51.65%
Xm169	96	52.75%
Xm17	106	58.24%
Xm101	138	75.82%
Xm209	82	45.05%
Xm99	96	52.75%
Xm89	91	50.00%
Xm225	145	79.67%

**Table 6:** Genetic diversity indices of 10 microsatellite DNA markers for the F_1_ population of *P. cantorii*.

Locus	2019 Generation							2020 Generation							Total Population						
	A	A_e_	I	H_o_	H_e_	PIC	HW	A	A_e_	I	H_o_	H_e_	PIC	HW	A	A_e_	I	H_o_	H_e_	PIC	HW
Xm86	4	3.747	1.353	0.854	0.737	0.684	***	4	3.346	1.291	0.760	0.706	0.650	NS	4	3.614	1.335	0.813	0.725	0.674	***
Xm207	2	1.844	0.650	0.359	0.460	0.353	NS	3	1.969	0.685	0.317	0.495	0.371	*	2	1.976	0.687	0.341	0.495	0.372	***
Xm106	2	1.606	0.565	0.505	0.379	0.306	*	2	1.672	0.592	0.532	0.404	0.321	NS	2	1.635	0.577	0.517	0.389	0.313	***
Xm169	2	1.793	0.634	0.583	0.444	0.344	*	2	1.624	0.573	0.468	0.387	0.310	NS	2	1.723	0.610	0.533	0.421	0.332	**
Xm17	2	1.999	0.693	0.631	0.502	0.375	NS	2	1.980	0.688	0.519	0.498	0.372	NS	2	1.994	0.692	0.582	0.500	0.374	NS
Xm101	2	1.920	0.672	0.796	0.482	0.364	***	2	1.844	0.650	0.709	0.461	0.353	***	2	1.890	0.667	0.758	0.472	0.360	***
Xm209	2	1.882	0.661	0.437	0.470	0.359	NS	2	1.921	0.673	0.468	0.482	0.365	NS	2	1.890	0.666	0.451	0.475	0.361	NS
Xm99	2	1.703	0.603	0.583	0.415	0.328	***	2	1.576	0.552	0.456	0.368	0.299	ND	2	1.649	0.583	0.528	0.395	0.316	***
Xm89	2	1.715	0.608	0.534	0.419	0.330	NS	2	1.640	0.579	0.456	0.393	0.314	NS	2	1.683	0.596	0.500	0.407	0.323	*
Xm225	5	3.044	1.199	0.806	0.675	0.607	***	3	2.635	1.033	0.785	0.624	0.550	NS	5	2.870	1.140	0.797	0.653	0.585	***
Average	2.5	2.125	0.764	0.609	0.498	0.405		2.3	2.021	0.731	0.547	0.482	0.3905		2.5	2.093	0.755	0.5819	0.493	0.401	

Abbreviations: A, number of alleles; A_e_, effective number of alleles; I, Shannon-Wiener Index; H_e_, expected heterozygosity; H_o_, observed heterozygosity; PIC, polymorphic information content; HW, Hardy–Weinberg equilibrium; NS, no deviation in HW; ND, no test results. * = *p*-value < 0.05; * ≥ 2 = *p*-value < 0.01.

**Table 7:** Allele frequency of 10 simple sequence repeat loci.

Xm86		Xm207		Xm106		Xm169		Xm17		Xm101		Xm209		Xm99		Xm89		Xm225	
A	F	A	F	A	F	A	F	A	F	A	F	A	F	A	F	A	F	A	F
173	0.1900	136	0.445	180	0.736	129	0.300	114	0.472	163	0.379	137	0.615	117	0.269	165	0.283	116	0.017
176	0.376	139	0.555	183	0.264	132	0.701	120	0.528	171	0.621	140	0.385	126	0.731	172	0.717	124	0.462
184	0.267																	126	0.003
190	0.168																	133	0.247
																		136	0.272

A, Allele; F, allele frequency.

**Table 8:** Inbreeding coefficients of 10 simple sequence repeat loci.

Xm86		Xm207		Xm106		Xm169		Xm17		Xm101		Xm209		Xm99		Xm89		Xm225	
A	F_IS_	A	F_IS_	A	F_IS_	A	F_IS_	A	F_IS_	A	F_IS_	A	F_IS_	A	F_IS_	A	F_IS_	A	F_IS_
173	0.198	136	0.310	180	0.330	129	0.270	114	0.168	163	0.611	137	0.048	117	0.341	165	0.232	116	0.017
176	0.169	139	0.310	183	0.33030	132	0.270	120	0.168	171	0.611	140	0.048	126	0.341	172	0.232	124	0.039
184	0.363																	126	0.003
190	0.201																	133	0.329
																		136	0.374
Total	0.124		0.310		0.330		0.270		0.168		0.611		0.048		0.341		0.232		0.223

A, Allele; F_IS_, inbreeding coefficient.

## Data Availability

The data presented in this study are available in this article and supplementary material.

## Supplementary Materials

The following supporting information can be downloaded at: https://www.mdpi.com/article/10.3390/ani12243459/s1, Figure S1: The original enomic DNA gel electrophoresis of some samples from *Pelochelys cantorii.*
