# Supplementary material for: Conservation Genetics of the Asian Giant Soft-Shelled Turtle (Pelochelys cantorii) with Novel Microsatellite Multiplexes
Source: Animals (Basel). 2022 Dec 8;12(24):3459. doi: 10.3390/ani12243459 (PMC9774628; doi:10.3390/ani12243459)
Supplement: Supplementary file 1 [file animals-12-03459-s001.zip › animals-1985453-supplementary.pdf]

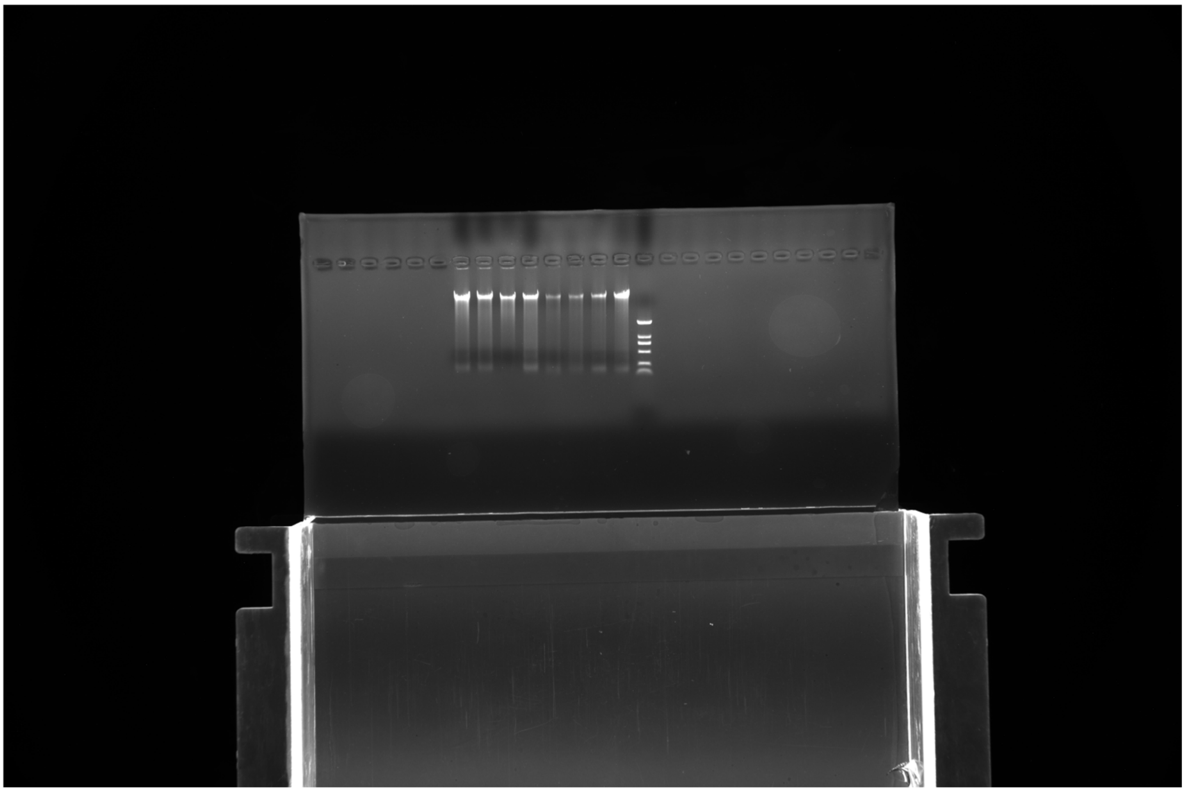

Figure S1. The original enomic DNA gel electrophoresis of some samples from *Pelochelys cantorii.*
